# The FIB-4 Index Is a Useful Predictor for the Development of Hepatocellular Carcinoma in Patients with Coexisting Nonalcoholic Fatty Liver Disease and Chronic Hepatitis B

**DOI:** 10.3390/cancers13102301

**Published:** 2021-05-11

**Authors:** Minah Kim, Yeonju Lee, Jun Sik Yoon, Minjong Lee, So Shin Kye, Sun Woong Kim, Yuri Cho

**Affiliations:** 1 Department of Family Medicine, National Police Hospital, Seoul 05715, Korea; mina11501@naver.com (M.K.); soskye@police.go.kr (S.S.K.); 2 Department of Internal Medicine, CHA Gangnam Medical Center, CHA University School of Medicine, Seoul 06135, Korea; yeonju1122@naver.com; 3 Department of Internal Medicine, Busan Paik Hospital, Inje University College of Medicine, Busan 47392, Korea; yojusi@naver.com; 4 Department of Internal Medicine, Ewha Womans University College of Medicine, Seoul 07804, Korea; minjonglee2@naver.com; 5 Center for Liver and Pancreatobiliary Cancer, National Cancer Center, Goyang 10408, Korea

**Keywords:** nonalcoholic fatty liver disease, chronic hepatitis B, hepatocellular carcinoma, FIB-4, predictor

## Abstract

**Simple Summary:**

This retrospective study analyzed 237 consecutive patients with coexisting nonalcoholic fatty liver disease and chronic hepatitis B (NAFLD-CHB) with long observation period (median follow-up duration, 13 years). The optimal cutoff for the FIB-4 index of 1.77 was calculated based on the maximum Youden index value, and the value was 1.77 with an AUC of 0.70. The significant higher risk of developing hepatocellular carcinoma (HCC) in patients with a high FIB-4 index (≥1.77) than the patients with a low FIB-4 index (<1.77) (adjusted hazard ratio, 4.35; 95% CI, 1.42–13.24; log-rank test, *p* = 0.006) were found among the NAFLD-CHB patients whose baseline characteristics were balanced by propensity score matching. The FIB-4 index might be a useful predictor of the development of HCC among NAFLD–CHB patients.

**Abstract:**

Background: The FIB-4 index, a noninvasive tool (FIB-4 index = age × aspartate transaminase (AST)/(platelet count × √alanine aminotransferase (ALT)), is a useful assessment for liver fibrosis. Patients with a high FIB-4 index were reported to have a high risk of developing hepatocellular carcinoma (HCC). This study analyzed the clinical association of the FIB-4 index with HCC development in patients with coexisting nonalcoholic fatty liver disease and chronic hepatitis B (NAFLD–CHB). Methods: This retrospective study analyzed 237 consecutive patients with NAFLD–CHB between January 2006 and December 2010 at the National Police Hospital in Korea. Patients with HCC at baseline and those diagnosed with HCC within 6 months from baseline were excluded. Propensity score matching analysis (PSM) was adopted to balance the baseline characteristics between patients with low and high FIB-4 index values. The cumulative rates of HCC development were compared between the two groups using the Kaplan–Meier method in the matched population. Results: The median follow-up duration was 13 years (interquartile range, 8.2–15.7). The optimal cutoff for the FIB-4 index of 1.77 was calculated based on the maximum Youden index value, with an AUC of 0.70. Among a total of 237 patients with NAFLD–CHB, HCC developed in 20 patients (8.4%) (14 of the 90 patients with a high FIB-4 index vs. 6 of the 147 patients (4.1%) with a low FIB-4 index; log-rank *p* = 0.003). Patients with a high FIB-4 index had a significantly and independently higher risk of HCC than those with a low FIB-4 index (adjusted hazard ratio, 4.35; 95%; confidence interval, 1.42–13.24; log-rank test, *p* = 0.006). Conclusion: A high FIB-4 index (≥1.77) might be a useful marker for predicting the development of HCC in patients with NAFLD–CHB.

## 1. Introduction

In 2020, hepatocellular carcinoma (HCC) was the sixth most common cancer and the third most common cause of cancer-related death worldwide [1]. HCC develops mainly in patients with risk factors such as nonalcoholic fatty liver disease (NAFLD), chronic hepatitis B (CHB), and chronic hepatitis C [2].

NAFLD is defined as an excessive accumulation of hepatic steatosis due to a cause other than alcohol consumption [3]. NAFLD is the most common liver disease and is estimated to have a 25% prevalence rate worldwide [4]. The prevalence of NAFLD is approximately 24% in North America, approximately 20–30% in Europe, and approximately 15–40% in Asia [5]. The number of patients with CHB is estimated to be between 240 million and 350 million, and it is particularly common in East Asia and sub-Saharan Africa [6]. As incidences of NAFLD are rapidly increasing, the number of patients with coexisting NAFLD and CHB (NAFLD–CHB) is also increasing [7].

The prognosis of NAFLD–CHB was reported to be worse than that of either NAFLD or CHB alone. In Asia, it was reported that patients with NAFLD–CHB had a 7.3-fold higher risk of developing HCC than CHB patients without NAFLD [8,9]. Similarly, in North America, CHB patients with nonalcoholic steatohepatitis (NASH) had a 1.7-fold higher risk of developing HCC than CHB patients without NASH [7]. Considering the increasing number of patients with NAFLD–CHB, and the fact that this population has a high risk of developing HCC, there is a need for a means of stratifying NAFLD–CHB patients according to their risk of developing HCC.

The FIB-4 index is a noninvasive tool (i.e., FIB-4 index = age × aspartate aminotransferase (AST)/platelet count × √alanine aminotransferase (ALT)) for assessing hepatic fibrosis [10]. The FIB-4 index is easy to use in clinical practice and has a comparable diagnostic capability for advanced fibrosis to that of magnetic resonance elastography [11]. Therefore, the FIB-4 index might be useful for the prediction of the risk of HCC. In previous studies, a high FIB-4 index was reported as a significant risk factor for developing HCC in patients with CHB [12,13,14] and patients with NAFLD [15,16].

In this study, we evaluated the clinical value of the FIB-4 index for the prediction of the development of HCC in patients with NAFLD–CHB.

## 2. Patients and Methods

### 2.1. Study Design

This study included consecutive patients with NAFLD–CHB seen between January 2006 and December 2010 at the National Police Hospital in Korea. Clinical, laboratory, and imaging data (i.e., abdominal ultrasonography) on the date of the initial visit between January 2006 and December 2010 were retrospectively reviewed. Laboratory evaluations included total bilirubin, AST, ALT, total cholesterol, glucose levels, and viral serology for hepatitis. Body mass index (BMI, weight (kg)/height (m)^2^), the hepatic-steatosis index score (HSI, 8 × ALT/AST ratio + BMI (+2 if diabetes mellitus; +2 if female)), and FIB-4 index were calculated at baseline [10,17]. Patients were classified as having NAFLD if they had sonographic findings characteristic of NAFLD and/or an HSI score >36. The use of antiviral medication for hepatitis B at baseline was defined as having a prescription within 1 year from baseline, and the use of antiviral agents after baseline was defined as having a prescription for more than a year during the observation period [12]. The use of aspirin or statins was defined by having a prescription for more than 30 days during the observation period.

All patients were monitored at 6-month intervals at minimum based on the international hepatitis B virus (HBV) guidelines for HCC surveillance [18,19]. The cumulative rates of HCC development were compared between the groups with high and low FIB-4 indices using the Kaplan–Meier method.

The development of HCC was diagnosed on the basis of the typical hallmarks of HCC: arterial phase hyperenhancement with washout in the portal venous or delayed phases on computed tomography and magnetic resonance imaging using contrast agents [20]. Patients were followed-up with until the date of diagnosis of HCC or the last visit before the data cutoff date, which was 31 December 2020.

This study was conducted in accordance with the guidelines of the Declaration of Helsinki and the principles of good clinical practice. The need to obtain informed consent was waived due to the retrospective design of the study, which was approved by the institutional review board (IRB No.11100176-202102-HR-002).

### 2.2. Patients

**Figure 1:**
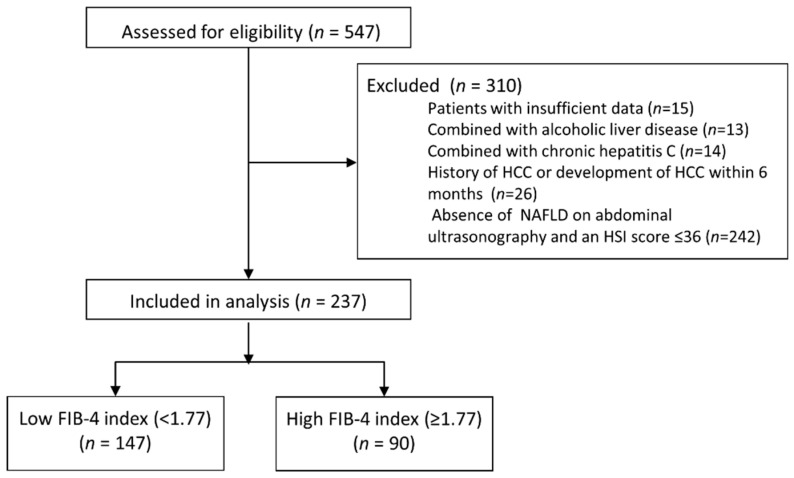
CONSORT diagram. Of the 547 eligible patients, 310 were excluded from the study in accordance with the inclusion and exclusion criteria. Finally, 237 patients were included: 147 in the low FIB-4 group (<1.77) and 90 patients in the high FIB-4 group (≥1.77).

The inclusion criteria for the study subjects were as follows: (i) the persistence of hepatitis B surface antigen (HBsAg) in serum samples for at least 6 months and (ii) the history of undergoing abdominal ultrasonography at baseline. A total of 547 patients satisfied the inclusion criteria. Among them, 310 patients were excluded based on the following exclusion criteria: (i) insufficient laboratory data (=15), (ii) significant alcohol intake (more than 60 g/day in both sexes; *n* = 13), (iii) combined with chronic hepatitis C (*n* = 14), (iv) hepatocellular carcinoma (HCC) at baseline or a diagnosis of HCC within 6 months after the date of enrollment (*n* = 26), and (v) absence of NAFLD on abdominal ultrasonography or an HSI score ≤36 (*n* = 242) (Figure 1) [21]. Finally, 237 patients were included in the study.

### 2.3. Statistical Analysis

The patients were divided into two groups according to the optimal cutoff value of the FIB-4 index for the prediction of the development of HCC. Patient baseline characteristics were compared between the two groups using the chi-square test or Fisher’s exact test for categorical variables and the Mann–Whitney *U* test for continuous variables. Propensity score matching (PSM) was performed to adjust potential confounders: sex, age, diabetes mellitus, ultrasonographic liver cirrhosis, BMI, history of drug use (i.e., antiviral agents at baseline, antiviral agents after baseline, aspirin, and statin), and HBV DNA levels [22]. The balance of the baseline characteristics between the two groups was re-evaluated after PSM.

To identify independent predictors of developing HCC, univariable Cox proportional hazard analysis and multivariable Cox proportional hazard analysis with backward stepwise regression were performed in the PSM cohort. Variables with *p* < 0.2 in univariable analysis were included in the multivariable analysis. If the variables in the multivariate analysis included two variables with a correlation coefficient greater than or equal to 0.4, only one variable with a lower *p*-value was included in the multivariable analysis. The correlation coefficients were calculated by using phi correlations between binary variables and biserial correlations between dichotomous and continuous variables. The development of HCC was assessed with Kaplan–Meier curves, and the two groups were compared with a log-rank test. All statistical analyses were performed using R software version 3.5.2 (http://www.r-project.org (accessed on 1 May 2021)). All tests were two-tailed, and *p*-values < 0.05 were considered significant.

### 2.4. Sensitivity Analysis

Two sensitivity tests were performed to support the results. First, the sensitivity analysis according to the change of the cutoff value of FIB-4 was performed among entire cohort (*n* = 237). Severe fibrosis (i.e.; F3-F4) increases the risk of developing HCC [23,24]. The minimum cutoff value of the FIB-4 index for F3-F4 was 1.3, and the maximum cutoff value was 2.67 [25,26,27]. The negative predictive value (NPV) of a FIB-4 cutoff value <1.3 was 90–95%, and the positive predictive value (PPV) of a FIB-4 cutoff value >2.67 was 80%. Based on previous studies on the FIB-4 index, we conducted a sensitivity analysis by dividing the patients into three groups as follows: FIB-4 index < 1.3, 1.3 ≤ FIB-4 index ≤ 2.67, and FIB-4 index > 2.67. For the analysis, one-way ANOVA and the Kruskal–Wallis test were used.

The second sensitivity analysis was performed to verify whether the results were changed according to the NAFLD diagnostic tool. The patients were classified as having NAFLD if they had sonographic findings characteristic of NAFLD.

## 3. Results

### 3.1. Baseline Characteristics

**Figure 2:**
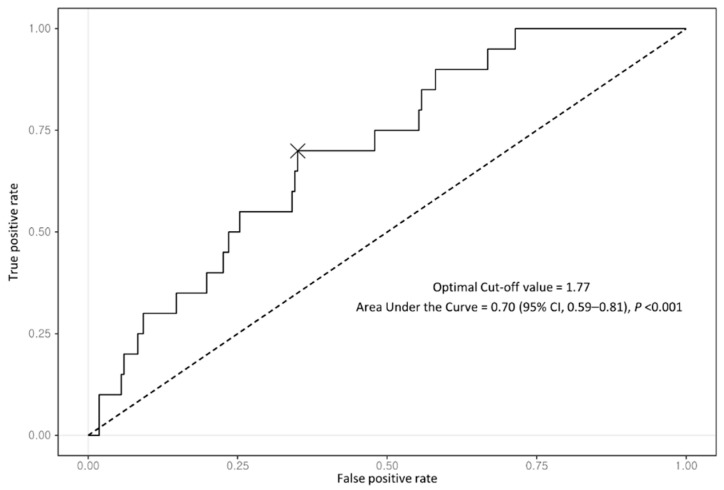
Receiver operating characteristic curve of FIB-4 index for predicting HCC development in entire cohort.

A total of 237 patients with coexisting NAFLD–CHB were included in this analysis. All patients showed positive HBsAg at baseline. Of them, 132 patients (55.7%) were treated with antiviral agents: the largest proportion of patients (72; 54.5%) took entecavir, followed by tenofovir disoproxil (29.5%), lamivudine (8.3%), adefovir (5.3%), and tenofovir alafenamide (2.3%) (Table 1). The median follow-up duration in the entire cohort was 13.0 years (interquartile range (IQR), 8.2–15.7 years). During the follow-up period, 20 patients (8.4%) were newly diagnosed with HCC. All patients were divided into two groups according to the optimal FIB-4 index cutoff, which had the best performance with regard to predicting the development of HCC based on the maximum Youden index value. The optimal cutoff for the FIB-4 index was 1.77 with an area under the curve of 0.70 (95% CI, 0.59–0.81) (Figure 2).

Among the entire cohort, 90 patients (38.0%) had a high FIB-4 index (≥1.77), and 147 patients (62.0%) had a low FIB-4 index (<1.77) at baseline. During the follow-up period, 14 patients (15.6%) in the high FIB-4 group developed HCC, and 6 patients (4.1%) in the low FIB-4 group developed HCC (log-rank test, *p* = 0.003). Overall, the median time to the development of HCC from baseline was 8.0 years (IQR, 5.9–10.2 years). There was no significant difference in the time to the development of HCC between the high FIB-4 group and the low FIB-4 group (8.0 years vs. 8.1 years, *p* = 0.72).

At baseline, there were no significant differences in the proportion of patients with HBeAg positivity and elevated serum HBV DNA levels (>2000 IU/mL) between the two groups. However, the proportion of patients using antiviral agents was significantly higher in the high FIB-4 group than in the low FIB-4 group (64.4% vs. 50.3%, *p* = 0.047).

After PSM, the baseline characteristics were well balanced between the high and low FIB-4 groups except the FIB-4 index components (Table 1). The variables associated with CHB, including the use of antiviral agents at baseline (36.7% vs. 46.7%, *p* = 0.23), HBeAg positivity (50.2% vs. 52.2%, *p* = 0.89), and serum HBV DNA levels (42.2% vs. 38.9%, *p* = 0.76), were not significantly different between the two groups. In contrast, variables used in the calculation of the FIB-4 index, namely, age (46.5 years vs. 51.5 years, *p* < 0.001), AST level (24.0 IU/L vs. 49.0 IU/L, *p* < 0.001), ALT level (34.5 IU/L vs. 63.5 IU/L, *p* < 0.001), and platelet count (193.0 × 10^9^/L vs. 156.5 × 10^9^/L, *p* = 0.001), were significantly different between the two groups.

There were two pairs of variables that showed a correlation coefficient greater than or equal to 0.4. The correlation coefficient between the use of antiviral agents at baseline and the use of antiviral agents after base line was 0.68; the correlation coefficient between the use of aspirin and the use of statin was 0.56 (Supplementary Figure S1).

### 3.2. Risk Factors for Developing HCC in NAFLD–CHB Patients

**Figure 3:**
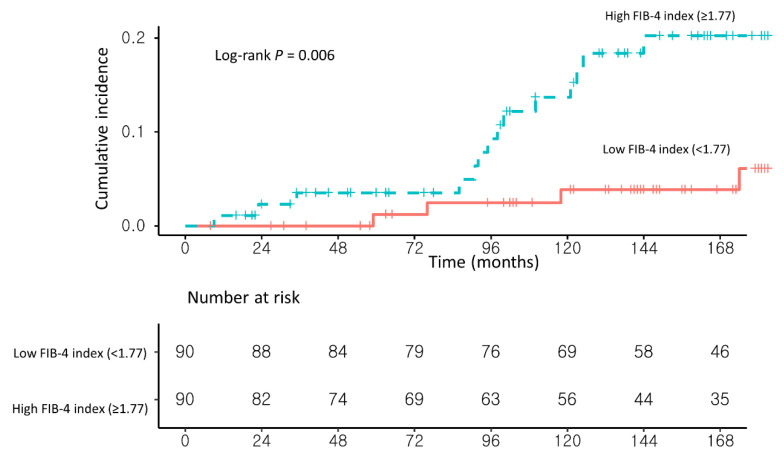
Propensity score-matched Kaplan–Meier estimates of HCC incidence in (**1**) NAFLD–CHB patients with a low FIB-4 index (<1.77) and (**2**) NAFLD–CHB patients with a high FIB-4 index (≥1.77).

In univariable analysis, a high FIB-4 index (≥1.77) and the presence of liver cirrhosis on USG (USG-LC) were significantly associated with the development of HCC. In multivariable Cox proportional hazard analysis with stepwise regression, patients with a high FIB-4 index (≥1.77) had a significantly higher risk of HCC development than those with a low FIB-4 index (<1.77) (adjusted hazard ratio (Ahr), 4.35; 95% confidence interval (CI), 1.42–13.24; log-rank test, *p* = 0.006; Table 2 and Figure 3).

### 3.3. Sensitivity Analysis

**Figure 4:**
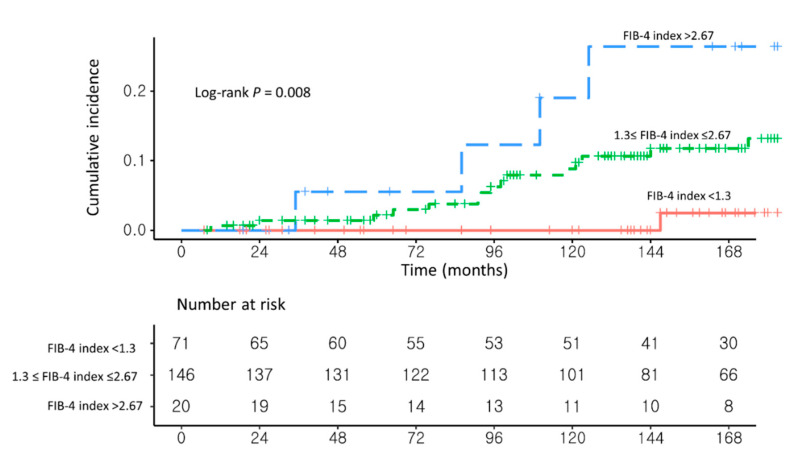
Unadjusted Kaplan–Meier estimates of HCC incidence in (**1**) NAFLD–CHB patients with FIB-4 index < 1.3, (**2**) NAFLD–CHB patients with 1.3 ≤ FIB-4 index ≤ 2.67, and (**3**) NAFLD–CHB patients with FIB-4 index > 2.67.

As a sensitivity analysis, the risk factors for HCC development were analyzed in three groups: FiB-4 index < 1.3 (*n* = 71), 1.3 ≤ FIB-4 index ≤ 2.67 (*n* = 146), and FIB-4 index > 2.67 (*n* = 20; Supplementary Table S1). Patients with a FIB-4 index >2.67 had a significantly higher risk of developing HCC than those with a FIB-4 index <1.3 (aHR, 12.51, 95% CI, 1.36–115.03; *p* = 0.02; Figure 4 and Supplementary Table S2). Similarly, patients with a 1.3 ≤ FIB-4 index ≤ 2.67 had a higher risk of developing HCC than those with a FIB-4 index <1.3 (aHR, 5.10; 95% CI, 0.67–38.88; *p* = 0.12).

**Figure 5:**
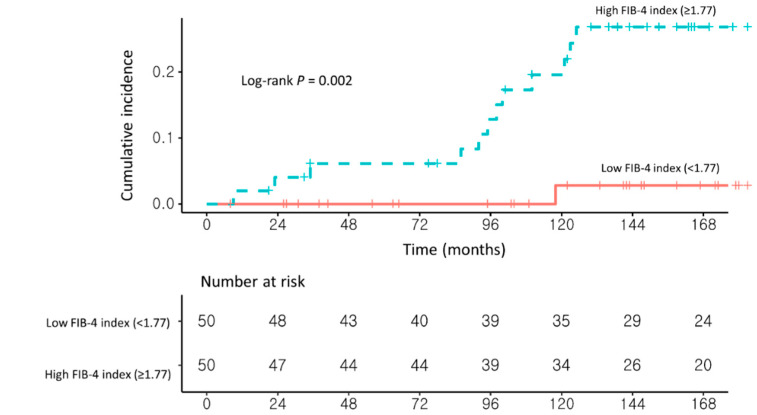
Propensity score-matched Kaplan–Meier estimates of HCC incidence in (**1**) NAFLD–CHB patients with a low FIB-4 index (<1.77) and (**2**) NAFLD–CHB patients with a high FIB-4 index (≥1.77) among ultrasonographic NAFLD patients.

In the second sensitivity analysis, 131 patients were classified as having NAFLD according to ultrasonographic findings. After PSM, patients with a high FIB-4 index (≥1.77; *n* = 50) had a significantly higher risk of HCC development than those with a low FIB-4 index (<1.77; *n* = 50; aHR, 20.59; 95% CI, 2.54–166.65; log-rank test, *p* = 0.002; Figure 5 and Supplementary Table S3).

## 4. Discussion

In this study, the risk of developing HCC was significantly higher in NAFLD–CHB patients with a high FIB-4 index (≥1.77) than in those with a low FIB-4 index (<1.77; aHR, 4.35; 95% CI, 1.42–13.24; log-rank *p* = 0.006). Analysis of long-term follow-up data of NAFLD–CHB patients showed that patients at a higher risk of developing HCC could be identified using the FIB-4 index, which is simple and easy to use in clinical practice.

We were able to estimate hepatic fibrosis with the FIB-4 index based on the patient’s age and the results of simple blood tests (i.e., the AST level, ALT level, and platelet count) [10]. Age, AST and ALT levels, and the platelet count are known to be associated with hepatic fibrosis [28,29,30]. Studies performed after the development of the FIB-4 index verified that the FIB-4 index can be used to identify advanced fibrosis in patients with NAFLD, CHB, or chronic hepatitis C [27,31,32,33,34]. Of course, the gold standard for the diagnosis of hepatic fibrosis is liver biopsy. However, considering the difficulty of performing invasive biopsies in all NAFLD–CHB patients, the FIB-4 index might be suitable as a clinical tool for the evaluation of hepatic fibrosis.

Hepatic fibrosis is a well-known risk factor for the development of HCC [35,36]. Therefore, we hypothesized that the risk of developing HCC could be predicted using the FIB-4 index. Recent studies have suggested a role of the FIB-4 index as a prognostic predictor in patients with CHB or NAFLD. Suh et al. reported that a high FIB-4 index had better predictive value for the development of HCC than USG-LC among patients with CHB [12]. Kanwal et al. also reported that NAFLD patients without cirrhosis and a low FIB-4 index had a significantly lower incidence of HCC than those with a high FIB-4 index (cutoff value = 2.67) [15].

In this study, NAFLD–CHB patients with a high FIB-4 index (≥1.77) had a higher risk of developing HCC than patients with a low FIB-4 index (<1.77). To verify this result, a sensitivity analysis was performed based on the cutoff values used in previous studies: FIB-4 index < 1.3, 1.3 ≤ FIB-4 index ≤ 2.67 (as the gray zone), and FIB-4 index > 2.67 [15,25,26,27]. Consistent with the results of the main analysis, patients with a FIB-4 index >2.67 had a significantly higher risk of developing HCC than patients with a FIB-4 index <1.3 (aHR, 12.51, 95% CI, 1.36–115.03, *p* = 0.02). The presence of the gray zone enabled the detection of a significant difference between the upper and lower groups.

This study stratified the risk of developing HCC in NAFLD–CHB patients, who are at an elevated risk of developing HCC [8,9]. Chan et al. reported that coexisting NAFLD was an independent risk factor for HCC development by 7.3-folds in CHB patients. Similarly, Lee et al. reported that coexisting NAFLD was associated with a 3-fold increased risk for HCC development in CHB patients. In CHB patients who with an already high risk of developing HCC, the mechanism by which NAFLD–CHB further increases the risk is unclear. Insulin resistance, which is common in patients with NAFLD, may increase the risk of developing HCC [37]. Under the influence of insulin resistance, increased free fatty acids promote intracellular oxidative stress and the production of reactive oxygen species (ROS). High ROS levels serially damage DNA and induce hypoadiponectinemia [38]. In one animal experiment, hypoadiponectinemia was associated with a risk of developing HCC [39]. Changes in gut microbiota or increased platelet activation may also influence hepatocarcinogenesis [40,41].

Among NAFLD–CHB patients, those with a high FIB-4 index (≥1.77) are at high risk for HCC. Surveillance should be based on the risk of developing HCC in the target population, which aims to reduce the patient’s HCC-related death [20]. Patients diagnosed with HCC at a very early stage can expect a 5-year survival rate of 8–90% through resection or ablation treatment [42], and patients diagnosed with HCC at an early stage can expect a 5-year survival rate of 5–60% through resection, liver transplantation or ablation treatment. On the other hand, patients diagnosed at an advanced stage who receive systemic therapies can expect median survival times of 6–8 months. As a tool for screening patients at high-risk of developing HCC, the use of the FIB-4 index in surveillance programs may contribute to improving the prognosis of NAFLD–CHB patients.

Several limitations of this study must be acknowledged. Because the present study was retrospective in nature, there were significant differences in baseline characteristics between the high FIB-4 index and the low FIB-4 index group. To overcome this limitation, we applied two statistical methods: a multivariable Cox proportional hazard model and PSM. In addition, since this was a study conducted on patients at the National Police Hospital in Korea, the cohort was predominantly male (90.3%), and since this study was only conducted in Korea, it did not reflect regional differences, especially between non-Asian countries and Asian countries.

In conclusion, over a mean 13-year follow-up period, patients with a high FIB-4 index (≥1.77) had a significantly higher risk of developing HCC than patients with a low FIB-4 index (<1.77). The FIB-4 index might be a useful predictor of the development of HCC among NAFLD–CHB patients. Large-scale prospective studies are needed in the future.

## Figures and Tables

**Table 1:** Baseline characteristics of the NAFLD–CHB patients with a low FIB-4 index (<1.77) and NALFD-CHB patients with a high FIB-4 index (≥1.77).

Variables		Before Propensity Score Matching			After Propensity Score Matching *		
	Total Population (*n* = 237)	Low FIB-4 Index	High FIB-4 Index	*p* Value	Low FIB-4 Index	High FIB-4 Index	*p* Value
		(*n* = 147)	(*n* = 90)		(*n* = 90)	(*n* = 90)	
Male (vs. female)	214 (90.3%)	137 (93.2%)	77 (85.6%)	0.09	83 (92.2%)	77 (85.6%)	0.24
Diabetes mellitus (yes vs. no)	55 (23.2%)	36 (24.5%)	19 (21.1%)	0.66	27 (30.0%)	19 (21.1%)	0.13
Hypertension (yes vs. no)	52(21.9%)	28 (19.0%)	24 (26.7%)	0.22	23 (25.6%)	24 (26.7%)	1.00
USG-LC (yes vs. no)	37 (15.6%)	18 (12.2%)	19 (21.1%)	0.1	16 (17.8%)	19 (21.1%)	0.85
Body mass index (kg/m^2^)	25.0 (25.0–26.8)	25.0 (25.0–26.6)	25.0 (25.0–27.2)	0.56	25.0 (24.7–26.3)	25.0 (25.0–27.2)	0.37
Antiviral treatment at baseline (yes vs. no)	84 (35.4%)	42 (28.6%)	42 (46.7%)	0.007	33 (36.7%)	42 (46.7%)	0.23
Antiviral treatment after baseline (yes vs. no)	132 (55.7%)	74 (50.3%)	58 (64.4%)	0.047	51 (56.7%)	58 (64.4%)	0.29
Aspirin use (yes vs. no)	42 (17.7%)	25 (32.9%)	17 (18.9%)	0.85	20 (22.2%)	17 (18.9%)	0.71
Statin use (yes vs. no)	49 (20.7%)	32 (21.8%)	17 (18.9%))	0.71	20 (22.2%)	17 (18.9%))	0.47
HBeAg positivity (yes vs. no)	121 (51.1%)	74 (50.3%)	47 (52.2%)	0.88	45 (50.0%)	47 (52.2%)	1
HBV DNA > 2000 IU/mL (yes vs. no)	96 (40.5%)	61 (41.5%)	140 (22.2%)	<0.001	38 (42.2%)	35 (38.9%)	0.65
Total bilirubin (IU/L)	0.8 (0.6–1.0)	0.8 (0.6–1.0)	0.8 (0.6–1.1)	0.26	0.8 (0.6–1.1)	0.8 (0.6–1.1)	0.23
Cholesterol (mg/dL)	171.0 (151.0–194.5)	172.0 (150.0–194.5)	168.0 (151.0–193.0)	0.77	168.0 (150.0–193.0)	168.0 (151.0–193.0)	0.82
FIB-4 index	1.6 (1.2–2.0)	1.3 (1.1–1.5)	2.2 (1.9–2.6)	<0.001	1.4 (1.2–1.6)	2.2 (1.9–2.6)	<0.001
Age (years)	46.0 (39.0–52.0)	43.0 (36.5–49.0)	51.5 (47.0–55.0)	<0.001	46.5 (42.0–52.0)	51.5 (47.0–55.0)	<0.001
AST (IU/L)	30.0 (23.0–46.0)	25.0 (20.5–31.0)	49.0 (36.0–67.0)	<0.001	24.0 (20.0–30.0)	49.0 (36.0–67.0)	<0.001
ALT (IU/L)	41.0 (28.0–70.0)	35.0 (26.0–51.0)	63.5 (35.0–94.0)	<0.001	34.5 (25.0–48.0)	63.5 (35.0–94.0)	<0.001
Platelet (10^9^/L)	182.0 (150.0–220.0)	197.0 (161.5–231.5)	156.5 (121.0–197.0)	<0.001	193.0 (155.0–224.0)	156.5 (121.0–197.0)	0.001

NOTE. Data are expressed as *n* (%) or median (interquartile range). USG-LC, ultrasonographic liver cirrhosis; HBV, hepatitis B virus; AST, aspartate aminotransferase; ALT, alanine aminotransferase. * Adjusted for sex, age, diabetes mellitus, ultrasonographic liver cirrhosis, BMI, history of drug use (i.e., antiviral agents at baseline, antiviral agents after baseline, aspirin, and statin), and HBV DNA levels.

**Table 2:** Risk factors of HCC development in the propensity score matched cohort.

Variables	Univariable Analysis		Multivariable Analysis	
	HR (95% CI)	*p* Value	aHR (95% CI)	*p* Value
Male (vs. female)	1.32 (0.17–9.99)	0.78	–	–
Age (years)	1.00 (0.95–1.07)	0.88	–	–
Diabetes mellitus (yes vs. no)	1.65 (0.62–4.41)	0.31	–	–
USG-LC (yes vs. no)	7.59 (2.94–19.59)	<0.001	7.84 (3.03–20.28)	<0.001
Body mass index ≥25 (vs. <25)	1.55 (0.61–3.93)	0.35	–	–
Antiviral treatment at baseline (yes vs. no)	0.65 (0.24–1.73)	0.39	–	–
Antiviral treatment after baseline (yes vs. no)	2.02 (0.67–6.15)	0.21	–	–
Aspirin use (yes vs. no)	0.21 (0.03–1.57)	0.13	–	–
Statin use (yes vs. no)	0.20 (0.02–1.54)	0.12	–	–
HBeAg positivity (yes vs. no)	0.93 (0.37–2.33)	0.87	–	–
HBV DNA >2000 IU/mL (yes vs. no)	0.63 (0.24–1.67)	0.35	–	–
High FIB-4 index (≥1.77) (vs. <1.77)	4.16 (1.37–12.65)	0.01	4.35 (1.42–13.24)	0.009

HCC, hepatocellular carcinoma; HR, hazard ratio; CI, confidence interval; aHR, adjusted hazards ratio; USG-LC, ultrasonographic liver cirrhosis; HBV, hepatitis B virus.

## Data Availability

Not applicable.

## Supplementary Materials

The following are available online at https://www.mdpi.com/article/10.3390/cancers13102301/s1, Figure S1: The heatmap of correlation coeffients among the propensity score matched cohort, Table S1: Baseline characteristics of the NAFLD-CHB patients with FIB-4 index < 1.3, the NAFLD-CHB patients with 1.3 ≤ FIB-4 index ≤ 2.67, and the NAFLD-CHB patients with FIB-4 index > 2.67, Table S2: Risk factors of HCC development in the entire cohort, and Table S3: Risk factors of HCC development in the propensity score matched cohort among ultrasonographic NAFLD patients.
