# Supplementary material for: The FIB-4 Index Is a Useful Predictor for the Development of Hepatocellular Carcinoma in Patients with Coexisting Nonalcoholic Fatty Liver Disease and Chronic Hepatitis B
Source: Cancers (Basel). 2021 May 11;13(10):2301. doi: 10.3390/cancers13102301 (PMC8151791; doi:10.3390/cancers13102301)
Supplement: Supplementary file 1 [file cancers-13-02301-s001.zip › cancers-1183535-supplementary.pdf]

Supplement

Supplementary Figure 1. The heatmap of correlation coeffients among the propensity score matched cohort **p. 2**

Supplementary Table 1. Baseline characteristics of the NAFLD-CHB patients with FIB-4 index <1.3, the NAFLD-CHB patients with 1.3≤ FIB-4 index ≤2.67, and the NAFLD-CHB patients with FIB-4 index >2.67 **p. 3**

Supplementary Table 2. Risk factors of HCC development in the entire cohort. **p. 5**

Supplementary Table 3. Risk factors of HCC development in the propensity score matched cohort among ultrasonographic NAFLD patients. **p. 7**

Supplementary Figure 1. The heatmap of correlation coeffients among the propensity score matched cohort

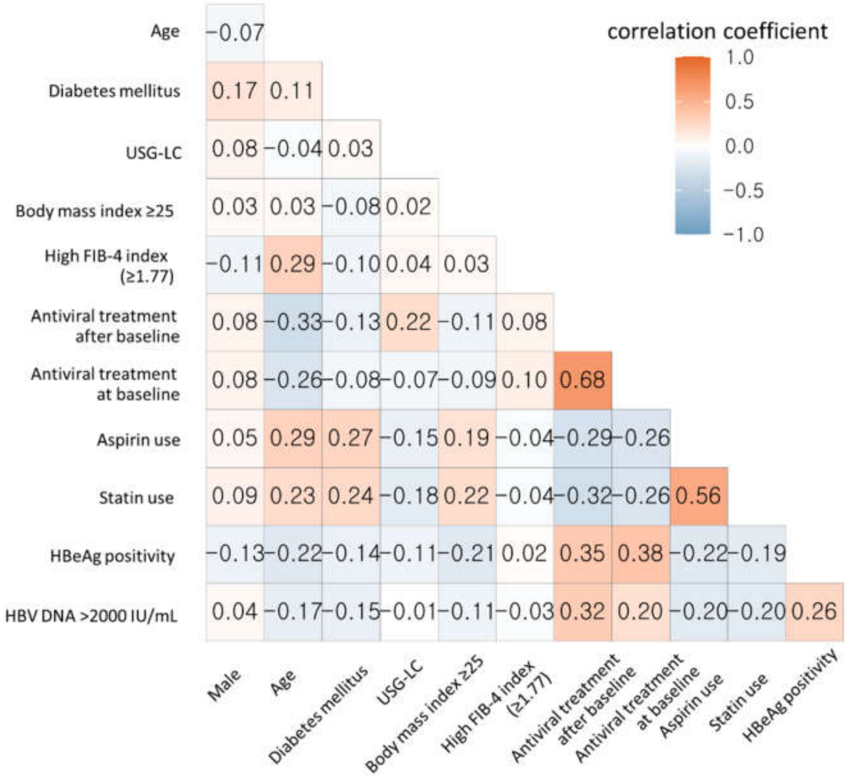

USG-LC, ultrasonographic liver cirrhosis; HBV, hepatitis B virus.

Supplementary table 1. Baseline characteristics of the NAFLD-CHB patients with FIB-4 index <1.3, the NAFLD-CHB patients with 1.3≤ FIB-4 index ≤2.67, and the NAFLD-CHB patients with FIB-4 index >2.67

Variables	FIB-4 index <1.3 (n=71)	1.3≤ FIB-4 index ≤2.67 (n=146)	FIB-4 index >2.67 (n=20)	*P* value
**Male (vs. female)**	68 (95.8%)	131 (89.6%)	15 (75.0%)	0.02
**Diabetes mellitus (yes vs. no)**	11 (15.5%)	39 (26.7%)	5 (25.0%)	0.18
**Hypertension (yes vs. no)**	6 (8.5%)	38 (26.0%)	8 (40.0%)	0.001
**USG-LC (yes vs. no)**	4 (5.6%)	26 (17.8%)	7 (35.0%)	0.002
**Body mass index (kg/m^{2})**	25.0 (25.0–26.2)	25.0 (25.0–26.8)	25.6 (25.0–28.5)	0.22
**Antiviral treatment at baseline (yes vs. no)**	25 (35.2%)	49 (33.6%)	10 (50.5%)	0.35
**Antiviral treatment after baseline (yes vs. no)**	41 (57.7%)	78 (53.4%)	13 (65.0%)	0.57
**Aspirin use (yes vs. no)**	5 (7.0%)	31 (21.2%)	6 (30.0%)	0.007
**Statin use (yes vs. no)**	12 (16.9%)	32 (21.9%)	5 (25.0%)	0.61
**HBeAg positivity (yes vs. no)**	43 (60.6%)	67 (45.9%)	11 (55.0%)	0.12

**HBV DNA >2000 IU/mL (yes vs. no)**	34 (47.9%)	55 (37.7%)	7 (35.0%)	0.31
**Total bilirubin (IU/L)**	0.8 (0.6–1.0)	0.8 (0.6–1.0)	0.9 (0.6–1.4)	0.32
**Cholesterol (mg/dL)**	176.0 (151.5–193.0)	170.5 (150.0–198.0)	166.5 (152.0–186.0)	0.70
**FIB-4 index**	1.1 (0.9–1.2)	1.7 (1.5–2.0)	3.1 (2.7–3.7)	<0.001
**Age (years)**	37.0 (34.0–41.0)	49.0 (45.0–53.0)	52.0 (45.0–61.0)	<0.001
**AST (IU/L)**	24.0 (20.0–31.0)	30.5 (25.0–47.0)	66.5 (51.0–100.0)	<0.001
**ALT (IU/L)**	39.0 (26.5–58.0)	41.0 (28.0–67.0)	74.0 (50.5–126.0)	0.006
**Platelet (10^9/L)**	200.0 (168.0–233.5)	182.0 (144.0–219.0)	131.5 (97.5–164.0)	<0.001

NOTE. Data are expressed as n (%) or median (interquartile range)

USG-LC, ultrasonographic liver cirrhosis; HBV, hepatitis B virus; AST, aspartate aminotransferase; ALT, alanine aminotransferase.

Supplementary table 2. Risk factors of HCC development in the entire cohort.

Variables	Univariable analysis		Multivariable analysis	
	HR (95% CI)	*P* value	aHR (95% CI)	*P* value
**Male (vs. female)**	1.26 (0.17–9.46)	0.82	–	–
**Age (years)**	1.02 (0.98–1.08)	0.26	–	–
**Diabetes mellitus (yes vs. no)**	2.07 (0.82–5.19)	0.12	2.12 (0.83–5.40)	0.11
**USG-LC (yes vs. no)**	9.03 (3.69–22.11)	<0.001	5.95 (2.39–14.82)	0.001
**Body mass index ≥25 (vs. <25)**	1.41 (0.58–3.40)	0.45	–	–
**Antiviral treatment at baseline (yes vs. no)**	0.74 (0.29–1.94)	0.54	–	–
**Antiviral treatment after baseline (yes vs. no)**	1.62 (0.62–4.22)	0.32	–	–
**Aspirin use (yes vs. no)**	0.22 (0.03–1.66)	0.14	–	–
**Statin use (yes vs. no)**	0.18 (0.02–1.32)	0.09	0.20 (0.03–1.56)	0.12
**HBeAg positivity (yes vs. no)**	0.83 (0.34–2.00)	0.68	–	–
**HBV DNA >2000 IU/mL (yes vs. no)**	0.55 (0.21–1.44)	0.22	–	–
**FIB-4 index <1.3**	1 [Reference]	–	1 [Reference]	–
**1.3≤ FIB-4 index ≤2.67**	7.15 (0.94–54.13)	0.06	5.10 (0.67–38.88)	0.12

**FIB-4 index >2.67**	16.18 (1.80–144.82)	0.01	12.51 (1.36–115.03)	0.02

HCC, hepatocellular carcinoma; HR, hazard ratio; CI, confidence interval; aHR, adjusted hazards ratio; UGS-LC, ultrasonographic liver cirrhosis; HBV, hepatitis B virus

Supplementary table 3. Risk factors of HCC development in the propensity score matched cohort among ultrasonographic NAFLD patients.

Variables	Univariable analysis		Multivariable analysis	
	HR (95% CI)	*P* value	aHR (95% CI)	*P* value
**Male (vs. female)**	1.23 (0.16–9.47)	0.84	–	–
**Age (years)**	1.02 (0.95–1.09)	0.58	–	–
**Diabetes mellitus (yes vs. no)**	2.54 (0.83–7.77)	0.10	6.37 (1.69–24.06)	0.006
**USG-LC (yes vs. no)**	10.66 (3.54–32.13)	<0.001	31.38 (7.53–130.65)	<0.001
**Body mass index ≥25 (vs. <25)**	0.06 (0.32–2.86)	0.94	–	–
**Antiviral treatment at baseline (yes vs. no)**	0.64 (0.19–2.09)	0.46	–	–
**Antiviral treatment after baseline (yes vs. no)**	2.47 (0.68–8.98)	0.16	–	–
**Aspirin use (yes vs. no)**	0.22 (0.03–1.71)	0.15	–	–
**Statin use (yes vs. no)**	0.23 (0.03–1.80)	0.16	–	–
**HBeAg positivity (yes vs. no)**	0.72 (0.24–2.13)	0.55	–	–
**HBV DNA >2000 IU/mL (yes vs. no)**	0.69 (0.22–2.11)	0.52	–	–

**High FIB-4 index (≥1.77). (vs. <1.77)**	12.13 (1.58–93.28)	0.02	20.59 (2.54–166.65)	0.004

HCC, hepatocellular carcinoma; HR, hazard ratio; CI, confidence interval; aHR, adjusted hazards ratio; USG-LC, ultrasonographic liver cirrhosis; HBV, hepatitis B virus.
